# Sustaining knowledge translation interventions for chronic disease management in older adults: protocol for a systematic review and network meta-analysis

**DOI:** 10.1186/s13643-018-0808-4

**Published:** 2018-09-15

**Authors:** Andrea C. Tricco, Julia E. Moore, Nicole Beben, Ross C. Brownson, David A. Chambers, Lisa R. Dolovich, Annemarie Edwards, Lee Fairclough, Paul P. Glasziou, Ian D. Graham, Brenda R. Hemmelgarn, Bev Holmes, Wanrudee Isaranuwatchai, Chantelle C. Lachance, France Legare, Jessie McGowan, Sumit R. Majumdar, Justin Presseau, Janet E. Squires, Henry T. Stelfox, Lisa Strifler, Kristine Thompson, Trudy Van der Weijden, Areti Angeliki Veroniki, Sharon E. Straus

**Affiliations:** 1 Li Ka Shing Knowledge Institute, St. Michael’s Hospital, Knowledge Translation Program, 209 Victoria Street, East Building, Room 716, Toronto, Ontario M5B 1W8 Canada; 2 Epidemiology Division, Dalla Lana School of Public Health, University of Toronto, 155 College Street, 6th floor, Toronto, Ontario M5T 3M7 Canada; 3 Canadian Partnership Against Cancer, 1 University Avenue, Suite 300, Toronto, Ontario M5J 2P1 Canada; 4 Prevention Research Center in St. Louis, Brown School, Washington University in St. Louis, St. Louis, MO 63130 USA; 5 Department of Surgery and Alvin J. Siteman Cancer Center, Washington University School of Medicine, Washington University in St. Louis, 660 S. Euclid Avenue, St. Louis, MO 63110 USA; 6 National Cancer Institute, 9609 Medical Center Drive, Room 3E414, Rockville, MD 20850 USA; 7 Leslie Dan Faculty of Pharmacy, University of Toronto, 144 College Street, Room 607, Toronto, Ontario M5S 3M2 Canada; 8 Department of Family Medicine David Braley Health Sciences Centre, McMaster University, 100 Main Street West, 5th floor, Hamilton, Ontario L8P 1H6 Canada; 9 Health Quality Ontario, 130 Bloor Street West, 10th floor, Toronto, Ontario M5S 1N5 Canada; 10 Faculty of Health Sciences and Medicine, Bond University, Robina, Queensland 4226 Australia; 11 School of Epidemiology and Public Health, University of Ottawa, 600 Peter Morand Crescent, Ottawa, K1G 5Z3 Canada; 12 The Ottawa Hospital Research Institute, 501 Smyth Road, Box 711, Ottawa, Ontario K1H 8L6 Canada; 13 Departments of Medicine and Community Health Sciences, University of Calgary, TRW Building, 3rd Floor, 3280 Hospital Drive NW, Calgary, Alberta T2N 4Z6 Canada; 14 The Michael Smith Foundation for Health Research (MSFHR), 200 - 1285 West Broadway, Vancouver, British Columbia V6H 3X8 Canada; 15 Département de Médecine Sociale et Préventive, Faculté de médecine, Université Laval Pavillon Ferdinand-Vandry1050, avenue de la Médecine, local 2431, Québec, Québec G1V 0A6 Canada; 16 Axe Santé des Populations et Pratiques Optimales en Santé, Centre de Recherche du CHU de Québec, 1050, chemin Sainte-Foy, local K0-03, Québec, Québec G1S 4L8 Canada; 17 Department of Medicine, University of Alberta, 13-103 Clinical Sciences Building, 11350-83 Avenue, Edmonton, Alberta T6G 2G3 Canada; 18 School of Nursing, University of Ottawa, 451 Smyth Road, Ottawa, Ontario K1H 8M5 Canada; 19 Departments of Critical Care Medicine, Medicine and Community Health Sciences, University of Calgary, 2500 University Drive NW, Calgary, Alberta T2N 1N4 Canada; 20 Department of Family Medicine, Maastricht University, CAPHRI Care and Public Health Research Institute, Universiteitssingel 40, 6229 ER Maastricht, The Netherlands; 21 Department of Geriatric Medicine, University of Toronto, 27 Kings College Circle, Toronto, Ontario M5S 1A1 Canada

**Keywords:** Sustainability, Knowledge translation, Chronic disease management, Older adults, Integrated knowledge translation

## Abstract

**Background:**

Failure to sustain knowledge translation (KT) interventions impacts patients and health systems, diminishing confidence in future implementation. Sustaining KT interventions used to implement chronic disease management (CDM) interventions is of critical importance given the proportion of older adults with chronic diseases and their need for ongoing care. Our objectives are to (1) complete a systematic review and network meta-analysis of the effectiveness and cost-effectiveness of sustainability of KT interventions that target CDM for end-users including older patients, clinicians, public health officials, health services managers and policy-makers on health care outcomes beyond 1 year after implementation or the termination of initial project funding and (2) use the results of this review to complete an economic analysis of the interventions identified to be effective.

**Methods:**

For objective 1, comprehensive searches of relevant electronic databases (e.g. MEDLINE, EMBASE, Cochrane Central Register of Controlled Trials), websites of health care provider organisations and funding agencies will be conducted. We will include randomised controlled trials (RCTs) examining the impact of a KT intervention targeting CDM in adults aged 65 years and older. To examine cost, economic studies (e.g. cost, cost-effectiveness analyses) will be included. Our primary outcome will be the sustainability of the delivery of the KT intervention beyond 1 year after implementation or termination of study funding. Secondary outcomes will include behaviour changes at the level of the patient (e.g. symptom management) and clinician (e.g. physician test ordering) and health system (e.g. cost, hospital admissions). Article screening, data abstraction and risk of bias assessment will be completed independently by two reviewers. Using established methods, if the assumption of transitivity is valid and the evidence forms a connected network, Bayesian random-effects pairwise and network meta-analysis will be conducted. For objective 2, we will build a decision analytic model comparing effective interventions to estimate an incremental cost-effectiveness ratio.

**Discussion:**

Our results will inform knowledge users (e.g. patients, clinicians, policy-makers) regarding the sustainability of KT interventions for CDM. Dissemination plan of our results will be tailored to end-users and include passive (e.g. publications, website posting) and interactive (e.g. knowledge exchange events with stakeholders) strategies.

**Systematic review registration:**

PROSPERO CRD42018084810

**Electronic supplementary material:**

The online version of this article (10.1186/s13643-018-0808-4) contains supplementary material, which is available to authorized users.

## Background

The failure to successfully sustain clinical interventions once implemented is a substantial global challenge with research waste estimated at > $200 billion per year [1], resulting in a need for relevant knowledge translation (KT) science (i.e. how to optimise research use in health care) and practice (i.e. implementing evidence in health care) to enhance return on research investments [2]. To optimise research impact and strengthen our health system, we need solutions (i.e. KT science) to the critical issue of how to sustain effective implementation (i.e. KT practice). Sustainability was identified as one of the most significant problems of our time, and failure to address it threatens health care globally [3].

To illustrate the issue, we consider older adults admitted to acute care hospitals: RCTs [4–6] show early mobilisation decreased their length of stay and improved their health. Early mobilisation is the clinical intervention; next, we need a tailored KT intervention (a strategy that facilitates research uptake in practice and policy) to implement it in hospitals; the KT interventions may target individuals (e.g. clinician audit and feedback, patient education) and organisations (e.g. policy for task shifting mobility to volunteers). Sixty-three Ontario hospitals used KT interventions [7] to implement an early mobilisation programme over 3 years and showed benefit; but, once effective KT interventions are in place, how do we sustain (and adapt) them? Despite a successful KT intervention, there is a tendency to return to old practices after initial KT activities end and attention and resources are turned to the next clinical issue [2]. Not only will this impact the KT intervention sustainability but it will also impact behaviour changes and health outcomes in patients; anyone who has tried to start exercising, quit smoking or lose weight knows how difficult it is to maintain their behaviour change, which may lead to negative health effects. Even if implementation has a small benefit, sustained implementation can have a major impact on patient outcomes over the long-term. Thus, failure to sustain KT interventions has implications for cost and patient care and diminishes confidence and support for future KT [8, 9]. While there is no uniform definition of KT sustainability [3], we define it as after a defined period of time, clinical and KT interventions continue to be delivered; clinician, patient, organisation behaviour change is maintained; and clinical and KT interventions may adapt, while continuing to produce benefits for individuals and systems [10].

Our overarcwhing research goal, identified by our knowledge users (e.g. clinicians, managers, patients, researchers), is to sustain implementation of effective clinical interventions through sustainable KT interventions to optimise care of older adults with chronic diseases. Adults aged ≥ 65 years are the largest growing proportion of our population and at increased risk of developing multiple chronic diseases (e.g. heart disease, dementia), translating to increased functional limitations, hospital admission and costs [11, 12]. There are safe and cost-effective chronic disease management (CDM) clinical interventions, but fewer than half of eligible patients receive them and significantly fewer sustain use (e.g. cardiac and osteoporosis treatment) [13], indicating a critical target for sustained KT interventions to optimise CDM. Indeed, given the proportion of older adults with chronic conditions and the expected growth in this population, KT interventions (targeting patients, clinicians, systems) offer solutions but often ignore real-world complexity (e.g. context and critical health determinants such as income, literacy and gender) and potential sustainability [3]. Our scoping review [14] of sustainability of CDM interventions identified 62 studies (> 260,000 patients) of 13 KT interventions (e.g. clinician reminders, patient motivational interviewing), indicating need for a systematic review and network meta-analysis (NMA; allowing comparison of multiple interventions and producing a ranked order of effectiveness using evidence from a network of trials). A NMA would identify effective and promising KT interventions among all available options. Furthermore, we did not include any economic studies in our scoping review [14]. To meet these challenges, our objectives are to (1) complete a systematic review and NMA of the comparative effectiveness and cost-effectiveness of sustainability of KT interventions that target CDM for end-users including older patients, clinicians, public health officials, health services managers and policy-makers on health care outcomes beyond 1 year or the termination of initial funding and (2) use the results of this review to complete an economic analysis of the interventions identified to be effective. Thus, we will identify strategies to assess and optimise sustainability of KT interventions for CDM in older adults, informing KT researchers and knowledge users about how to sustain implementation, by answering the question: What is the comparative effectiveness and cost-effectiveness of sustainability of knowledge translation interventions for chronic disease management in older adults?

## Methods/design

### Knowledge user engagement

Knowledge users, including patients, health care managers, policy-makers, KT researchers and KT practitioners are on the study team. The knowledge users have provided input into the research question and protocol and will be engaged throughout the research enterprise through monthly updates and quarterly webinars to discuss progress. The knowledge users have links to broad networks of potential collaborators who will be engaged around dissemination of the research results. We will assess stakeholder engagement through completion of the Patient and Public Engagement Questionnaire at study end, which has good construct validity (Dr. Ainsley Moore, personal communication).

### Objective 1

#### Protocol

A protocol for our systematic review was developed using the Preferred Reporting Items for Systematic Reviews and Meta-analyses Protocols (PRISMA-P) [15] to guide the reporting of the systematic review protocol (see Additional file 1). We registered the protocol with the PROSPERO database (CRD42018084810) [16].

#### Eligibility criteria

We will include randomised controlled trials (RCTs) with a comparator group examining the impact of a KT intervention targeting CDM in older adults after more than 1 year of implementation or after the termination of research/project funding is described. To examine cost, all economic studies (e.g. cost studies with a relevant comparator, cost-effectiveness analyses) will be included.

The target population for the CDM intervention includes patients (aged 65 years and older with one or more chronic disease including noncommunicable diseases) [17] or their caregiver. End-users of the KT intervention will include patients aged 65 years and older with one or more chronic disease, their caregivers, clinicians (from all disciplines), public health officials (including medical officers of health, department chairs, programme managers), health care managers and policy-makers (including regional, state/provincial, federal).

The CDM intervention is the clinical intervention such as use of exercise in a patient with type 2 diabetes; the KT intervention is the strategy used to support implementation of the CDM intervention, such as a reminder system for patients to exercise or motivational interviewing for clinicians to promote patient exercise. CDM interventions may target any chronic condition and may target the patient, caregiver, clinician or health system, with a goal to optimise health and well-being of the patient. All comparators are eligible for inclusion including other KT interventions or usual care.

Our primary outcome will be sustained implementation of the KT intervention beyond 1 year after implementation or the termination of funding as this was determined by our knowledge users to be relevant for decision-making in their contexts. Additionally, as secondary outcomes, we will consider sustained delivery of the CDM interventions as well of any patient (e.g. quality of life), clinician (e.g. eye examination frequency) or health care system (e.g. hospital admission) outcomes resulting from sustained behaviour change. Studies in all settings will be eligible, including primary and specialist care; acute and long-term care; inpatient and outpatient care; and regional, national, and international settings. Both published and unpublished material will be included, as well as those disseminated in any language.

#### Charting and outcome selection

The outcomes and outcome measures reported in all identified studies will be charted in Excel. All knowledge users (patients, clinicians, health care managers, policy-makers) on the team will then review this list and select the outcomes that are most relevant for their decision-making. We will aim to identify equal numbers of participants from each KU group, and in the case of non-response KUs will be asked to nominate an individual to replace them on the panel. Specifically, a modified Delphi approach [18] commonly used in quality improvement research [19] with two rounds will be used to achieve consensus on what KT and patient, clinician and health system outcomes should be considered for inclusion. First, an online survey using Qualtrics [20] will be sent to the knowledge users, which will include the list of outcomes, their definitions, and the frequency with which they appeared in the studies. The knowledge users will be invited to rate the importance of each outcome on a 7-point Likert scale ranging from ‘not at all important’ to ‘extremely important’. Second, their ratings will be aggregated and median ratings will be calculated. Outcomes with median ratings of ≥ 5 and appropriate levels of agreement among KUs (e.g. ≥ 75% of ratings must fall within three points of the median score) will be considered important outcomes and will be included in the second round. Third, in the second round, the knowledge users will assess the shortened list of preferred outcomes and will be asked to rank their top 3 outcomes. This process will be used to identify the primary and secondary outcomes by selecting the three outcomes with the highest ranking.

#### Information sources

We will conduct a systematic search of the published and difficult to locate or unpublished (i.e. grey) literature within health. Health will be defined using the World Health Organization (WHO) definition [21]. The following electronic databases will be searched from inception onwards: MEDLINE, EMBASE, Cochrane Central Register of Controlled Trials, Cumulative Index to Nursing and Allied Health Literature (CINAHL) and Campbell databases. A search of the grey literature will be conducted using a strategically developed Google search strategy, using guidance from the Canadian Agency for Drugs and Technologies in Health Grey Matters tool [22]. We will search the websites of key funding agencies (e.g. Canadian Institutes of Health Research) and health care provider organisations from Australia, Canada, the UK and the USA who have similar health care systems or similar challenges related to CDM. References from included studies and relevant articles will be scanned to ensure literature saturation. Team members will use their linkages with experts in the field to identify additional articles.

#### Search strategy

The main (i.e. MEDLINE) search strategy was developed by an experienced librarian, circulated to the team and revised, as necessary. The search used for our previous scoping review on this same topic was used as the basis for this search [23]; however, it was substantially modified to include various categorisations of potential KT interventions including the Effective Practice and Organisation of Care (EPOC) taxonomy of health systems interventions [24]. The search was organised according to the research question and PICO and included both MeSH and keyword terms for chronic medical diseases, specific MeSH and keyword terms for EPOC-related concepts and concepts related to knowledge translation and sustainability. The search was limited to humans and validated filters were used for older adults and randomised trials [25].

The search for the current review was then peer reviewed by a second experienced librarian using the Peer Review of Electronic Search Strategies (PRESS) Checklist [26]. The final search strategy can be found in Additional file 2. The search was adjusted and translated for other databases, as necessary, and these other search strategies are available from the authors upon reasonable request.

#### Study selection

The eligibility criteria will be pilot-tested on a random sample of 50 titles and abstracts from the literature search. All team members will screen these citations using the eligibility criteria independently and conflicts will be discussed. The eligibility criteria will be revised if deemed necessary by the team or if low agreement (< 90%) is observed. Two team members will then screen each citation independently using Synthesi.SR [27]. Similarly, we will conduct a calibration exercise of the eligibility criteria prior to screening potentially relevant full-text articles, which will then be screened by two team members independently. At both the citation and full-text levels of screening, conflicts will be resolved via discussion among pairs or reviewers or with a third member, if required.

#### Data items and data collection process

Two investigators will independently read each article and extract relevant data when available. We will abstract data at the following levels:

1. Study level: study design, year of study conduct, sample size, setting, country of study conduct, study funding source and KT theory/model/framework used to inform study
2. Patient level: type and number of patients, age mean and standard deviation, proportion of female participants, type of chronic condition(s) and number of clusters
3. Intervention level: type (e.g. KT interventions will be classified using a behaviour change techniques taxonomy) [28], frequency and duration of CDM and KT interventions, provider and target
4. Outcome level: patient, clinical and health system including cost-effectiveness (e.g. incremental cost-effectiveness ratios (ICERs) cost per quality adjusted life year (QALY))

Prior to data abstraction, we will calibrate our data collection form on a random sample of five full-text articles. Each team member will extract the data, and the team will meet to discuss conflicts. The data collection form will be revised for clarity, as needed. Subsequently, two team members will conduct all data collection for each study independently. Study authors will be contacted for further information as needed when considering studies for inclusion and when conducting data abstraction. When multiple studies report data from the same study population, the study with the longest follow-up and available data will be considered the main publication and the others will be retained for supplementary material only.

Two reviewers with expertise in KT and research methods will independently code each KT intervention first using the pre-existing taxonomy developed by the Cochrane EPOC group and then a behaviour change technique taxonomy to identify the specific active components in each intervention [24, 28]. Conflicts in KT intervention coding will be resolved through discussion. We will use this coding structure to create the nodes for the NMA by ‘lumping’ interventions according to the taxonomy categories and have used this approach in other NMAs of complex interventions [29].

#### Methodological quality/risk of bias appraisal

Two reviewers will conduct risk of bias independently on each included study. If there is disagreement, a third reviewer will be available. The risk of bias of RCTs will be done using the Cochrane EPOC Risk of Bias tool [30], and cost-effectiveness analysis studies will be evaluated against the reporting checklist developed by Drummond and colleagues [31]. For outcomes reported in 10 or more studies, small-study effects (e.g., publication bias) will be assessed using comparison-adjusted funnel plots [32].

#### Synthesis of included studies

We will describe the study characteristics, patient characteristics, outcome results, the methodological quality and risk of bias. We will report the results using the PRISMA extension for NMAs [33].

We anticipate clinical and methodological heterogeneity and thus will conduct random-effects meta-analysis in a Bayesian framework. We will assess heterogeneity of the included studies in terms of clinical (e.g. patient characteristics), methodological (e.g. study design) and statistical (e.g. heterogeneity in study outcomes between studies) characteristics. For example, clinicians on the team will assess clinical heterogeneity and methodologists will assess study heterogeneity. Statistical heterogeneity will be assessed by visual inspection of each meta-analysis forest plot, by estimating the between-study variance and by using the *I*^2^ statistic [25, 34]. If extensive heterogeneity is observed, we will try to explain this via sub-group analysis and meta-regression analysis [35].We will use vague priors for all model parameters aside from the between-study variance for which we will use the informative priors suggested by Turner and colleagues for dichotomous data and by Rhodes and colleagues for continuous data [36, 37]. In each NMA, we will assume a common within-network between-study variance across treatment comparisons and we will use an informative prior as suggested in Turner et al. [36], Rhodes et al. [37] and Nikolakopoulou et al. [38]. Multi-arm studies will be included in a head-to-head meta-analysis using the exact adjustment method as described by Rucker et al. [39]. The meta-regression analyses will be conducted when 10 or more studies are available for the underlying outcome and intervention comparison and will examine the influence of factors such as age and severity of chronic disease and risk of bias on the meta-analysis results.

For continuous outcomes, to make use of all data, we will impute missing measures of variance using established methods [40]. To ensure our imputations do not bias our results, we will conduct a sensitivity analysis to examine the missing data under random, completely at random and non-random assumptions.

The transitivity assumption will be assessed visually to ensure that potential effect modifiers (e.g. patient age, comorbidities) are balanced on average across comparisons [41]. We expect that transitivity will be valid if the common intervention used to compare different KT interventions indirectly is similar when it appears in different trials. Consistency of the entire network will be assessed with the design-by-treatment interaction model [42]. If inconsistency is found within the network, the loop-specific approach will be used to assess local inconsistency of the loops within each network [43]. If the assumption of transitivity is valid and the evidence forms a connected network, we will conduct a NMA using a Bayesian random-effects model.

Across all analyses, the summary intervention effects will be presented using the odds ratio or mean difference with the corresponding 95% credible intervals. Predictive intervals will also be reported for meta-analyses and NMA point estimates. We will assess model convergence using the Gelman-Rubin diagnostic and goodness of model fit [44] will be assessed using the posterior residual deviance, the degree of heterogeneity, and the Deviance Information Criterion (DIC) [45]. A well-fitting model has a residual deviance close to the number of data points. A difference of ≥ 3 units in DIC is considered important and the lowest DIC value implies the best fitting model. Mean ranks and the surface under the cumulative ranking curves (SUCRAs) will be used to derive a relative ranking of the KT interventions based on the NMA results [46]. Rank-heat plots will be used to display the intervention rankings across multiple outcomes [47].

Network subgroup and meta-regression analyses will be done to explore potential effect modifiers that impact transitivity or consistency assumptions when ten or more studies are available in the underlying outcome. If there are a sufficient number of studies, we will explore the following potential effect modifiers: sex, type of chronic disease, cluster of diseases (as reported in the studies such as diabetes and depression), severity of chronic diseases, care setting, and duration of follow-up. Sensitivity analysis will be done whereby we use data from the following studies in the NMA: (1) studies at low risk of bias based on the two components of our risk of bias assessment found to be of the greatest threat to study validity and (2) studies with high participant retention. Since the NMA is dependent on different priors for variance parameters included in the Bayesian approach, we will conduct a sensitivity analysis using vague priors [48]. All analyses will be performed within OpenBUGS, except for the consistency assessment that will be conducted in Stata using the *network* command [49, 50]. To ensure reproducibility, code listings or citations providing guidance on the methods employed in this NMA will be available upon request.

### Objective 2

#### Economic analyses

Using the findings from the systematic review and NMA, we will identify effective and sustainable KT interventions. Subsequently, we will conduct an economic evaluation to compare the cost and outcome among these KT interventions and use the results from the review to guide the selection of perspective, cost items to include, outcomes of interest and time horizon including other relevant model parameters. The details on specific data required for economic analyses will depend on the systematic review and NMA findings. Thus, a detailed protocol outlining the specific methods for the economic analysis will be prepared at a later date when NMA findings are available. We will conduct the analysis using the guideline from the Canadian Agency for Drugs and Technologies in Health [51] and based on the consolidated reporting standards [52].

## Discussion and dissemination

The sustainability of KT interventions is critical to ensuring long-term, high quality of care for patients, and this is particularly important for older adults with chronic diseases [53]. KT interventions that are not sustained represent an inefficient use of limited resources may worsen patient outcomes and negatively impact our health systems [13, 54]. As such, evaluating sustainability of KT interventions is increasingly important and sustainability has been identified as one of the critical gaps in KT science [3].

Our research will be relevant to KT researchers, as well as clinicians, patients and policy-makers. Our team includes representatives of these various knowledge user groups, and this will facilitate use of the research results. Indeed, we developed this protocol in direct response to needs identified by the knowledge users to address sustainability in health care. We will ensure that knowledge users continue to be engaged throughout the conduct of this review and they will lead dissemination efforts for our research results. We will use evidence-based approaches to dissemination, which will be tailored to the end-user needs. We will use a variety of passive (e.g. publication) and active (e.g. knowledge exchange events) dissemination strategies. We anticipate using the results of this review to inform the development of a toolkit for those interested in developing sustainable KT interventions for CDM in older adults.

## Additional files

Additional file 1: PRISMA-P 2015 Checklist. Preferred Reporting Items for Systematic Review and Meta-Analysis Protocols (PRISMA-P) 2015 statement. (DOCX 30 kb)

Additional file 2: MEDLINE Search Strategy. MEDLINE search strategy. (DOCX 23 kb)

## Abbreviations

CDM: Chronic disease management
CINAHL: Cumulative Index to Nursing and Allied Health Literature
DIC: Deviance Information Criterion
EPOC: Effective Practice and Organisation of Care
ICERs: Incremental cost-effectiveness ratios
KT: Knowledge translation
NMA: Network meta-analysis
PRESS: Peer Review of Electronic Search Strategies
PRISMA-P: Preferred Reporting Items for Systematic Reviews and Meta-analyses Protocols
QALY: Quality adjusted life year
RCTs: Randomised controlled trials
SUCRAs: Surface under the cumulative ranking curves
WHO: World Health Organization

## References

[1] Chalmers I, Glasziou P (2009). Avoidable waste in the production and reporting of research evidence. Lancet.

[2] Straus SE, Tetroe J, Graham ID (2013). Knowledge translation in health care: moving from evidence to practice.

[3] Proctor E, Luke D, Calhoun A, McMillen C, Brownson R, McCrary S (2015). Sustainability of evidence-based healthcare: research agenda, methodological advances, and infrastructure support. Implement Sci.

[4] Schweickert WD, Pohlman MC, Pohlman AS, Nigos C, Pawlik AJ, Esbrook CL (2009). Early physical and occupational therapy in mechanically ventilated, critically ill patients: a randomised controlled trial. Lancet.

[5] Fisher SR, Kuo YF, Graham JE, Ottenbacher KJ, Ostir GV (2010). Early ambulation and length of stay in older adults hospitalized for acute illness. Arch Intern Med.

[6] Chandrasekaran S, Ariaretnam SK, Tsung J, Dickison D (2009). Early mobilization after total knee replacement reduces the incidence of deep venous thrombosis. ANZ J Surg.

[7] Liu B, Moore JE, Almaawiy U, Chan WH, Khan S, Ewusie J, et al. Outcomes of Mobilisation of Vulnerable Elders in Ontario (MOVE ON): a multisite interrupted time series evaluation of an implementation intervention to increase patient mobilisation. Age Ageing. 2017:1–7. 10.1093/ageing/afx128 PMC5859974 28985310

[8] Evans-Lacko S, Jarrett M, McCrone P, Thornicroft G (2010). Facilitators and barriers to implementing clinical care pathways. BMC Health Serv Res.

[9] White DE, Norris JM, Jackson K, Khandwala F (2016). Barriers and facilitators of Canadian quality and safety teams: a mixed-methods study exploring the views of health care leaders. J Healthc Leadersh.

[10] Moore JE, Mascarenhas A, Bain J, Straus SE (2017). Developing a comprehensive definition of sustainability. Implement Sci.

[11] Hoffman C, Rice D, Sung HY (1996). Persons with chronic conditions. Their prevalence and costs. JAMA.

[12] Wodchis Walter P., Austin Peter C., Henry David A. (2016). A 3-year study of high-cost users of health care. Canadian Medical Association Journal.

[13] McGlynn EA, Asch SM, Adams J, Keesey J, Hicks J, DeCristofaro A (2003). The quality of health care delivered to adults in the United States. N Engl J Med.

[14] Tricco AC, Ashoor HM, Cardoso R, MacDonald H, Cogo E, Kastner M, et al. Sustainability of knowledge translation interventions in healthcare decision-making: a scoping review. Implement Sci. 2016;11:55. 10.1186/s13012-016-0421-7 PMC4839064 27097827

[15] Shamseer L, Moher D, Clarke M, Ghersi D, Liberati A, Petticrew M (2015). Preferred reporting items for systematic review and meta-analysis protocols (PRISMA-P) 2015: elaboration and explanation. BMJ.

[16] Tricco A, Moore J, Graham I, Isranuwatchai W, Presseau J, Squires J, et al. Sustaining knowledge translation interventions for chronic disease management in older adults: protocol for a systematic review and network meta-analysis. PROSPERO 2018 CRD42018084810 Available from: http://www.crd.york.ac.uk/PROSPERO/display_record.php?ID=CRD42018084810. Accessed 7 Feb 2018. 10.1186/s13643-018-0808-4 PMC6138921 30219107

[17] Noncommunicable diseases. http://www.who.int/ncds/introduction/en/. Accessed 29 Jan 2018.

[18] Dalkey N, Helmer O (1963). An experimental application of the DELPHI method to the use of experts. Manag Sci.

[19] Boulkedid R, Abdoul H, Loustau M, Sibony O, Alberti C (2011). Using and reporting the Delphi method for selecting healthcare quality indicators: a systematic review. PLoS One.

[20] Qualtrics - Qualitative Survey Software. https://www.qualtrics.com. 25 Oct 2017.

[21] Constitution of WHO: principles. http://www.who.int/about/mission/en/. 15 Jan 2018.

[22] Grey Matters: a practical tool for searching health-related grey literature. https://www.cadth.ca/resources/finding-evidence/grey-matters. 25 Oct 2017.

[23] Tricco AC, Cogo E, Ashoor H, Perrier L, McKibbon KA, Grimshaw JM, et al. Sustainability of knowledge translation interventions in healthcare decision-making: protocol for a scoping review. BMJ Open. 2013;3. https://www.ncbi.nlm.nih.gov/pubmed/?term=23674448. 10.1136/bmjopen-2013-002970 PMC3657660 23674448

[24] EPOC Taxonomy. http://epoc.cochrane.org/epoc-taxonomy. 25 Oct 2017.

[25] Cochrane Handbook for Systematic Reviews of Interventions. http://training.cochrane.org/handbook. 25 Oct 2017.

[26] McGowan J, Sampson M, Salzwedel DM, Cogo E, Foerster V, Lefebvre C (2016). PRESS peer review of electronic search strategies: 2015 guideline statement. J Clin Epidemiol.

[27] Newton D (2012). Synthesi.SR.

[28] Presseau J, Ivers NM, Newham JJ, Knittle K, Danko KJ, Grimshaw JM (2015). Using a behaviour change techniques taxonomy to identify active ingredients within trials of implementation interventions for diabetes care. Implement Sci.

[29] Tricco AC, Thomas SM, Veroniki AA, Hamid JS, Cogo E, Strifler L (2017). Comparisons of interventions for preventing falls in older adults: a systematic review and meta-analysis. JAMA.

[30] Cochrane EPOC Risk of Bias tool. http://epoc.cochrane.org/sites/epoc.cochrane.org/files/uploads/Risk%20of%20Bias%2005-01-2009.doc. 25 Oct 2017.

[31] Drummond MF, Sculpher MJ, Torrance GW, O'Brien BJ, Stoddart GL (2005). Methods for the economic evaluation of health care programme.

[32] Chaimani A, Higgins JP, Mavridis D, Spyridonos P, Salanti G (2013). Graphical tools for network meta-analysis in STATA. PLoS One.

[33] Hutton B, Salanti G, Caldwell DM, Chaimani A, Schmid CH, Cameron C (2015). The PRISMA extension statement for reporting of systematic reviews incorporating network meta-analyses of health care interventions: checklist and explanations. Ann Intern Med.

[34] Higgins JPT, Thompson SG, Deeks JJ, Altman DG (2003). Measuring inconsistency in meta-analyses. Br Med J.

[35] Meta-regression Approaches. https://www.ahrq.gov/research/findings/evidence-based-reports/metaregression.html. 25 Oct 2017.

[36] Turner RM, Davey J, Clarke MJ, Thompson SG, Higgins JP (2012). Predicting the extent of heterogeneity in meta-analysis, using empirical data from the Cochrane Database of Systematic Reviews. Int J Epidemiol.

[37] Rhodes KM, Turner RM, Higgins JP (2015). Predictive distributions were developed for the extent of heterogeneity in meta-analyses of continuous outcome data. J Clin Epidemiol.

[38] Nikolakopoulou A, Chaimani A, Veroniki AA, Vasiliadis HS, Schmid CH, Salanti G (2014). Characteristics of networks of interventions: a description of a database of 186 published networks. PLoS One.

[39] Rucker G, Cates CJ, Schwarzer G (2017). Methods for including information from multi-arm trials in pairwise meta-analysis. European J Combin.

[40] Furukawa TA, Barbui C, Cipriani A, Brambilla P, Watanabe N (2006). Imputing missing standard deviations in meta-analyses can provide accurate results. J Clin Epidemiol.

[41] Li T, Puhan MA, Vedula SS, Singh S, Dickersin K (2011). Network meta-analysis-highly attractive but more methodological research is needed. BMC Med.

[42] White IR, Barrett JK, Jackson D, Higgins JP (2012). Consistency and inconsistency in network meta-analysis: model estimation using multivariate meta-regression. Res Synth Methods.

[43] Veroniki AA, Vasiliadis HS, Higgins JP, Salanti G (2013). Evaluation of inconsistency in networks of interventions. Int J Epidemiol.

[44] Gelman A, Rubin DB (1996). Markov chain Monte Carlo methods in biostatistics. Stat Methods Med Res.

[45] Spiegelhalter DJ, Best NG, Carlin BP, van der Linde A (2002). Bayesian measures of model complexity and fit. J R Stat SocSer B Stat Methodol.

[46] Salanti G, Ades AE, Ioannidis JP (2011). Graphical methods and numerical summaries for presenting results from multiple-treatment meta-analysis: an overview and tutorial. J Clin Epidemiol.

[47] Veroniki AA, Straus SE, Fyraridis A, Tricco AC (2016). The rank-heat plot is a novel way to present the results from a network meta-analysis including multiple outcomes. J Clin Epidemiol.

[48] Lambert PC, Sutton AJ, Burton PR, Abrams KR, Jones DR (2005). How vague is vague? A simulation study of the impact of the use of vague prior distributions in MCMC using WinBUGS. Stat Med.

[49] Making BUGS Open. https://cran.r-project.org/doc/Rnews/Rnews_2006-1.pdf. 25 Oct 2017.

[50] Palmer T, Sterne J (2016). Meta-analysis in Stata: an updated collection from the Stata journal, 2nd ed.

[51] Guidelines for the Economic Evaluation of Health Technologies: Canada. https://www.cadth.ca/about-cadth/how-we-do-it/methods-and-guidelines/guidelines-for-the-economic-evaluation-of-health-technologies-canada. 25 Oct 2017.

[52] Husereau D, Drummond M, Petrou S, Carswell C, Moher D, Greenberg D (2013). Consolidated health economic evaluation reporting standards (CHEERS)--explanation and elaboration: a report of the ISPOR health economic evaluation publication guidelines good reporting practices task force. Value Health.

[53] Scheirer MA (2005). Is sustainability possible? A review and commentary on empirical studies of program sustainability. Am J Eval.

[54] Glasziou Paul, Straus Sharon, Brownlee Shannon, Trevena Lyndal, Dans Leonila, Guyatt Gordon, Elshaug Adam G, Janett Robert, Saini Vikas (2017). Evidence for underuse of effective medical services around the world. The Lancet.

