# Supplementary material for: Sustaining knowledge translation interventions for chronic disease management in older adults: protocol for a systematic review and network meta-analysis
Source: Syst Rev. 2018 Sep 15;7:140. doi: 10.1186/s13643-018-0808-4 (PMC6138921; doi:10.1186/s13643-018-0808-4)
Supplement: Supplementary file 2 — MEDLINE Search Strategy. MEDLINE search strategy. (DOCX 23 kb) [file 13643_2018_808_MOESM2_ESM.docx]

Additional File 2

# MEDLINE Search Strategy

Database: Ovid MEDLINE(R) Epub Ahead of Print, In-Process & Other Non-Indexed Citations, Ovid MEDLINE(R) Daily and Ovid MEDLINE(R) <1946 to Present>

Search Strategy:
--------------------------------------------------------------------------------
1     exp Chronic Disease/
2     ((chronic or longterm or long-term) adj (disease*or illness* or condition*)).tw,kw.
3     chronic* ill*.tw,kw.
4     exp *Angina Pectoris/
5     (stenocardia* or angina).ti.
6     exp *Cardiovascular Diseases/
7     ((cardiovascular or cardio-vascular) adj (disease* or abnormalit* or infection*)).ti.
8     (aneurysm* or aortic rupture* or endoleak* or microaneurysm* or hypertension or high blood pressure* or heart disease* or heart failure or cardiac failure or cardiac edema* or cardia oedema* or cardiac arrest*).ti.
9     (arrhythmia* or bradycardia* or tachycardia* or cardiac dysrhythmia* or heart block* or ventricular fibrillation* or atrial fibrillation*).ti.
10     (cardiomegaly or hypertrophy or enlarged heart or heart enlarge*).ti.
11     (cardiomyopath* or cardio-myopath* or myocarditis or myocardial disease* or endomyocardial fibrosis).ti.
12     (thrombosis or thrombus or thromboses or thromboembolism* or embolism* or thrombophlebitis* or lemierre syndrome).ti.
13     (peripheral vascular disease* or peripheral angiopath* or peripheral arterial disease* or phlebitis or raynaud disease*).ti.
14     (arteriosclerosis or atherosclerosis or atheromata* or atherogenesis).ti.
15     (coronary disease* or myocardial infarct* or heart attack*).ti.
16     exp *Cerebrovascular Disorders/
17     ((cerebrovascular or cerebro-vascular or vascular) adj (disorder* or disease* or occlusion*)).ti.
18     (ischemia or stroke or strokes or carotid artery disease* or arterial disease* or vascular dementia or arteriovenous malformation*).ti.
19     (intracranial h?emorrhage* or cerebral h?emorrhage* or cerebrovascular trauma* or vascular headach* or central nervous system vasculitis or Intracranial vasospasm* or brain infarc*).ti.
20     exp *Ischemia/
21     exp *Arthritis/
22     (arthritis or osteoarthritis or osteo-arthritis or gout or caplan syndrome or sjogren* syndrome or rheumatoid nodule or rheumatoid vasculitis or still* disease* or periarthritis or rheumatic fever or spondylarthritis).ti.
23     exp *Osteoporosis/
24     Osteoporosis.ti.
25     exp *Asthma/
26     (asthma or asthmas).ti.
27     exp *Neoplasms/
28     (carcinoma* or neoplasm* or tumor* or tumour* or cancer* or adenocarcinoma* or malignanc* or ependymoma* or leukemia* or lymphoma* or sarcoma* or rhabdomyosarcoma* or osteosarcoma* or adenoma* or adenosarcoma* or carcinosarcoma* or blastoma* or nephroma* or thymoma* or melanoma* or retinoblastoma*).ti.
29     exp *Pulmonary Disease, Chronic Obstructive/
30     (COPD or chronic obstructive pulmonary disease* or chronic bronchitis or pulmonary emphysema*).ti.
31     exp *Inflammatory Bowel Diseases/
32     *Irritable Bowel Syndrome/
33     (ulcerative colitis or crohn* disease* or inflammatory bowel disease* or irritable bowel syndrome* or mucous colitis).ti.
34     exp *Diabetes Mellitus/
35     (diabetes or diabetic or IDDM or wolfram syndrome* or T1DM or T2DM or T1 DM or T2 DM or NIDDM).ti.
36     exp *Mental Disorders/
37     exp *Sleep Wake Disorders/
38     ((drug or substance or alcohol or opioid or fentanyl or marijuana or cocaine or tobacco or amphetamine) adj (addiction* or abus* or dependenc* or disorder*)).ti.
39     (depression* or depressive disorder* or anxiet* or dementia).ti.
40     (sleep* adj2 disorder*).ti.
41     exp *HIV Infections/
42     (acquired immunodeficiency syndrome* or AIDS arteritis or AIDS virus or AIDS related or AIDS dementia or HIV infection* or human immunodeficiency virus* or HIV seropositivit* or HIV wasting syndrome*).ti.
43     or/1-42
44     Caregivers/
45     (care giver* or caregiver* or care next giver* or carer* or companion* or client care attendant* or home-care assistant* or home-care worker* or home care worker* or home-care aide* or home care aide* or home health care worker* or home healthcare worker* home health care assistant* or home healthcare assistant* or home health care aide* or home healthcare aide* or homemaker* or home-maker* or housekeeper* or house-keeper* or home support worker* or home health aid* or home visitor* or in-home assistant* or personal aide* or personal assistant* or personal care aide* or personal care attendant* or personal care provider* or personal support aide* or personal support worker* or respite worker* or personal attendant*).tw,kw.
46     44 or 45
47     43 and 46
48     aged.sh. or age*.tw.
49     43 and 48
50     limit 43 to "all aged (65 and over)"
51     47 or 49 or 50
52     (randomized controlled trial or controlled clinical trial or pragmatic clinical trial).pt.
53     (randomized or placebo or randomly).tw. or trial.ti.
54     clinical trials as topic/
55     or/52-54
56     51 and 55
57     animals/ not humans/
58     56 not 57
59     Waiting Lists/
60     (wait* list* or queu*).tw,kw.
61     exp *"Continuity of Patient Care"/
62     (care continuity or "coordination of care" or shared care).tw,kw.
63     ((continui* or continuum*) adj2 (care or healthcare)).tw.
64     Triage/
65     (triage* or triaging).tw,kw.
66     *Quality Assurance, Health Care/
67     exp *Quality Improvement/
68     *total Quality Management/
69     (quality assurance or total quality management or TQM or continuous quality improvement or CQI or QI).tw,kw.
70     (quality adj (improvement$ or initiativ$ or intervention$ or program$ or plan$ or audit$)).tw,tw.

71 outreach.tw,kw.
72     exp Self Care/
73     *Self Efficacy/
74     exp Self Help Devices/
75     (self car* or self-car* or self help or self-help or self manag$ or self-manag$ or self-monitor* or self monitor* or goal setting or self efficacy or self-efficacy).tw,kw.
76     exp *social support/
77     (social network* or social support* or social systems*).tw,kw.
78     ((length or time) adj3 (consult* or consultation*)).tw,kw.
79     exp *"Referral and Consultation"/
80     models, nursing/
81     "Personnel Staffing and Scheduling"/og [Organization & Administration]
82     (staff* adj (model or models)).tw,kw.
83     exit interview*.tw,kw.
84     Personnel Turnover/ and (Interviews as Topic/ or interview*.tw,kw.)
85     Personnel Selection/ and (exp Rural Health Services/ or Medically Underserved Area/)
86     ((recruit* or hire or hiring or retention or retain* or train* or retrain* or re-train* or personnel select* or staff select*) adj2 (underserv* or rural or physician shortage* or doctor shortage* or health worker* or health manager* or health system manager*)).tw,kw.
87     district health manager*.tw,kw.
88     or/59-87
89     58 and 88
90     exp Patient Care Planning/
91     exp disease management/
92     (advance care planning or care pathway* or case management or case coordination or case co-ordination or disease management or patient care management or patient care planning or patient handoff or patient hand-off or patient discharge or discharge plan* or care pathway*).tw,kw.
93     Geriatric Assessment/
94     (geriatric assessment or psychogeriatric* or psycho-geriatri* or gerontolog* assessment*).tw,kw.
95     exp Interprofessional Relations/
96     (interdisciplinary communication* or interprofessional communication* or interdisciplinary relation* or interprofessional relation* or inter-disciplinary communication* or inter-professional communication* or inter-disciplinary relation* or inter-professional relation*).tw,kw.
97     Communication/ and exp Health Personnel/
98     (communication adj2 (provider* or manager* or professional* or health personnel or health care personnel or physician* or doctor* or nurse* or pharmacist* or medical staff* or health care worker* or health worker* or specialist*)).tw,kw.
99     ("package of care" or (package* adj care)).tw,kw.
100     delivery of health care/mt
101     exp "Appointments and Schedules"/ and Patient Participation/
102     ((Patient initiated or patient-initiated) adj2 (follow up or follow-up or appointment* or schedul*)).tw,kw.
103     Patient Participation/ and (exp "Appointments and Schedules"/ or follow up.tw,kw. or follow-up.tw,kw. or appointment*.tw,kw. or schedule*.tw,kw.)
104     "Referral and Consultation"/og [Organization & Administration]
105     (referral system* or referral software*).tw,kw.
106     (shared adj2 care).tw,kw.
107     Decision Making/
108     (shared decision making or shared decision-making).tw,kw.
109     patient transfer/
110     ("transfer of care" or patient transfer*).tw,kw.
111     (guided care nurs* or transition coach* or nurse case manage* or advanced practice nurs* or care coordinator* or transition liaison nure* or nurse discharge advocate* or care transition nurs* or case manager* or transitional care nurs* or Home care nurs* or Liaison nurs* or Community care nurs* or System navigator* or Hospital home care coordinator* or Bed utilization coordinator* or Clinical nurse specialist* or Discharge coordinator* or Discharge planner* or Outreach coordinator* or Care coordinato* or Community case manager* or Patient flow coordinator* or Outreach nurs* or Outreach case manag* or Mental health case manag* or Community care coordinator* or Access homecare coordinato*r or Long term care transition* or elderly care transition*).tw,kw.
112     or/90-111
113     58 and 112
114     *Information Systems/
115     Health Information Systems/
116     Integrated Advanced Information Management Systems/
117     exp Management Information Systems/
118     "information and communication technolog*".kw,tw.
119     (health adj3 information system*).kw,tw.
120     (smart home* or smart environment*).kw,tw.
121     exp telemetry/
122     exp Telemedicine/
123     (telemedicine or telepathology or telerehabilitation or teleradiology or telenurs* or mobile health or ehealth or Tele-medicine or tele-pathology or tele-rehabilitation or tele-radiology or tele-nurs*).kw,tw.
124     Absenteeism/ and exp policy/
125     (absenteeism* and polic*).tw,kw.
126     exp *Accreditation/ or accreditation/ or accreditation.tw,kw.
127     Patient Rights/
128     (patient* adj right*).tw,kw.
129     or/114-128
130     58 and 129
131     Organizational Culture/ or organizational culture*.tw,kw. or organisational culture*.tw,kw. or corporate culture*.tw,kw.
132     Organizational Innovation/
133     "audit and feedback".tw,kw.
134     ((audit or audits or auditing) adj2 feedback).tw,kw.
135     (Medical Errors/ or Risk Management/ or incident report.tw,kw.) and (system*.tw,kw. or exp information systems/)
136     ("communities of practice" or "community of practice" or "practice community" or "practice communities").tw,kw.
137     ((education* adj2 gam*) or (education* adj2 play)).tw,kw.
138     exp Education/ and exp "Play and Playthings"/
139     exp Teaching Materials/
140     exp Inservice Training/
141     (education* adj2 (printed or material* or meeting* or outreach or visit* or conference* or workshop* or train* or inservice or in-service or cours*)).tw,kw.
142     exp Education, Continuing/
143     ((continuing or physician* or provider* or professional* or clinician* or doctor* or nurs* or pharmac*) adj2 (CME or educat* or train* or retrain* or re-train* or workshop* or professional development*)).tw,kw.
144     academic detail*.tw,kw.
145     practice guidelines as topic/
146     guideline adherence/
147     ((guideline* or CPG or CPGs) adj3 (disseminat$ or adherence)).tw,kw.
148     (consensus adj (expert or local or develop* or conference* or process* or workshop*)).tw,kw.
149     (group adj (nominal or technique* or process* or consensus)).tw,kw.
150     opinion leader*.tw,kw.
151     ((opinion or education* or influential) adj leader*).tw,kw.
152     manag* supervis*.tw,kw. or *Nursing, Supervisory/
153     patient mediat*.tw,kw.
154     (Patient Participation/ or Consumer Participation/) and (Professional Practice/ or exp Professional Competence/ or performance.tw,kw.)
155     (performance data adj5 public).tw,kw.
156     Reminder Systems/
157     (recall adj2 system*).tw.
158     (reminder* adj2 (prompt or system* or process* or manual or computer* intervention*)).tw,kw.
159     patient reported outcome measure*.tw,kw.
160     patient reported outcome measures/
161     (tailor* adj3 intervention*).tw,kw.
162     or/131-161
163     58 and 162
164     89 or 113 or 130 or 163
165     Translational Medical Research/
166     exp *Evidence-Based Practice/
167     *Program Evaluation/
168     *Information Dissemination/
169     Knowledge Management/
170     *"Outcome and Process Assessment (Health Care)"/
171     "process assessment (health care)"/
172     "Outcome Assessment (Health Care)"/
173     exp "diffusion of innovation"/
174     *Program Development/
175     (knowledge adj2 (translation or disseminat* or diffus* or exchange or transfer*)).tw,kw.
176     sustain*.tw,kw.
177     ((adherence or maintenance or adoption or adaptation or effect* or report or reporting or improv* or implement*) adj3 (innovation* or intervention* or strateg* or outcome* or process*)).tw,kw.
178     (diffusion adj3 innovation*).tw,kw.
179     (continued adj2 effect*).tw,kw.
180     or/165-179
181     164 and 180
182     (editorial or comment).pt.
183     181 not 182

**Economics and costs filters**
184     "Costs and Cost Analysis"/
185     (cost* or cost benefit analys* or cost effective*).tw.
186     Cost-Benefit Analysis/
187     Health Care Costs/
188     or/184-187

189     88 or 112 or 129 or 162 – EPOC sets
190     51 and 189 pre RCT line + EPOC sets
191     188 and 190 pre RCT line + EPOC sets + economics filter
192     180 and 191 pre RCT line + EPOC sets + economics filter + sustaining KT
**193     192 not 183 NOT RCT results**
194     animals/ not humans/
195     193 not 194
